# Factors which influence the consumption of street foods and fast foods in South Africa-a national survey

**DOI:** 10.1186/1475-2891-10-104

**Published:** 2011-10-04

**Authors:** Nelia P Steyn, Demetre Labadarios, Johanna H Nel

**Affiliations:** 1 Centre for the Study of Social and Environmental Determinants of Nutrition, Population Health, Health Systems and Innovation: Human Sciences Research Council, Cape Town, RSA; 2 Department of Logistics, University of Stellenbosch, Stellenbosch, Western Cape, RSA

## Abstract

**Background:**

Very little is known about street food and fast food consumption patterns in South Africa despite this being a large sector of the national economy in terms of employment provided and sales of food. The objective of this study was to determine the use of street foods and fast foods purchased by South Africans living in different provinces and geographic areas.

**Methods:**

A cross-sectional survey was conducted. Structured interview-administered questionnaires in 11 official languages were conducted at the participants' homes. A nationally representative sample (n = 3287) was drawn from all ethnic groups, and provinces including participants 16 years and older. Logistic regression was done to evaluate factors impacting on fast food consumption.

**Results:**

Frequent (2 ≥ times/week) street food consumption ranged from 1.8% in Northern Cape to 20.6% in Limpopo; frequent (2 ≥ times/week) fast food consumption ranged between 1.5% in North West Province to 14.7% in Gauteng. The highest intake of street food was in the medium socio-economic category (14.7%) while the highest intake of fast foods was in the high socio-economic category (13.2%). Overall, fruit was the most commonly purchased street food by all ethnic groups over the previous week although this practice was highest in black participants (35.8%). Purchases of soft drinks ranged from 4.8% in whites to 16.4% in blacks and savoury snacks from 2.3% to 14.5% in whites and blacks, respectively. Consumption of fast foods and street foods were influenced by a number of socio-demographic factors including ownership of major home appliances. Frequent fast food consumers had a significantly higher dietary diversity score (4.69; p < 0.0001) while frequent street food consumers had a significantly lower score (3.81; p < 0.0001).

**Conclusions:**

A large percentage of the population purchase street foods and fast foods. This is of some concern when one notes the high prevalence of soft drink consumption in terms of its association with obesity and non-communicable diseases. These findings need to be taken into consideration when evaluating dietary patterns and nutritional adequacy of population diets.

## Introduction

Very little is known about street food and fast food consumption in South Africa despite this being a large sector of the national economy in terms of employment provided and sales of food [1]. In South Africa, so-called "street foods" are regarded as being foods or beverages that are sold by the informal sector. Street foods are generally sold from stands/stalls (usually not permanent structures) on the pavement of busy streets in both urban and rural areas, usually at a lower cost than fast foods. Hence they provide an accessible source of food to poorer people. Generally only a few food items and beverages are for sale and many vendors sell the same items. Often these items include snacks such as crisps and drinks such as soft drinks; however cooked foods are also sold, frequently on site. Fast foods on the other hand are sold from outlets in formal structures such as buildings and malls and frequently operate as a franchise.

In 2009, Feeley et al., published data on fast food consumption (including street food sold by vendors) among 17 year olds in the *Birth to Twenty *cohort in Soweto and Johannesburg [2]. They found that 30% of the participants consumed fast foods five to seven times a week with another 20% having it two to four times a week. They postulated that street foods would probably make a significant contribution to total dietary intake since many items were substantial meals in terms of energy value, particularly the "Sowetan quarter (kota)" which comprises a quarter loaf of white bread with fried potato chips as the two main ingredients.

Another recent study undertaken at three different shopping malls in Johannesburg included young adults from three different socio-economic strata [3]. In this study only fast foods from retail outlets were included and not street foods. Almost half of the participants earned less than R5 000 ($714.29) per month yet spent at least R200 ($28.57) a month on fast foods. Twenty-eight percent had fast foods two to three times a month. The most popular food items were burgers, pizza and fried chicken, while soft drinks were the most common beverage consumed. The latter food items are a cause for alarm when one takes the high prevalence of obesity in South Africa into account (27.4% women are obese [4]), since the fast foods consumed were predominantly high-fat and/or energy-dense foods.

There is a paucity of data on street foods sold in South Africa, particularly with regard to types, frequency of use and contribution to nutritive value of the diet. This has long been a common practice in many African countries and contributes significantly to the employment of vendors, particularly women [5-7]. With the exception of the study undertaken in Soweto [2], information on street foods is virtually non-existent in South Africa. Furthermore, relatively little is known about fast food consumption in South Africa [2-4], and no published data is available at the national level.

The purpose of the current study was to evaluate and describe both street foods and fast foods bought by South Africans at the national level in order to determine whether this practice is likely to contribute significantly to dietary intake of the population and to make recommendations regarding a future research agenda in this regard. Furthermore, factors influencing fast food and street food purchasing were also evaluated.

## Methods

Since 2003 the Human Sciences Research Council (HSRC) has been conducting annual surveys on people's attitudes to and opinions on various viewpoints. The 2009 annual survey was designed to yield a nationally representative sample of participants aged 16 years and older. The Master Sample, which was designed in 2006, consisted of 1 000 primary sampling units. These units were drawn, with probability proportional to size, from a pre-census 2001 list of enumerator areas (EAs) provided by Statistics South Africa. In order to collect data for censuses, Statistics South Africa demarcates work areas that are manageable for one enumerator to enumerate within limited number of days, within the local municipalities and place names. Such areas are called EAs and are the units for planning, executing and capturing of census data. This spatial set of EA boundaries is updated before each census. The Master Sample focused on dwelling units or visiting points as secondary sampling units which were defined as residential stands, addresses, structures, flats and homesteads.

The sample was stratified to include all nine provinces (Table 1) and geographic areas (formal urban, informal urban, formal rural, tribal) and ethnic groups [black (African origin), white (Caucasian), mixed ancestry (European-African-Malayan decent), and Indian (original ancestors from India). The *tribal areas *refer to predominantly rural areas where traditionally chiefs still make decisions on matters under their jurisdiction. These areas include mostly those people living in the former TBVC homelands. TBVC refers to Transkei, Bophuthatswana, Venda and Ciskei. *Urban informal areas *include housing structures (often shacks) which are unplanned and usually do not have access to services such as electricity and water as opposed to *formal urban areas *which are structured and organized. A local council or district council controls development in these areas. Services such as water, electricity and refuse removal are provided; roads are formally planned and maintained by the council. This category includes suburbs, towns and townships [8].

**Table 1:** Percentage moderate and frequent purchasing of street foods and fast foods by province, gender and socio-economic category in South Africa

		**Street food Moderate**^**+ **^**use % (CI)**	**Street food Frequent**^**++**^ use % (CI)	**Fast foods Moderate**^**+ **^**use % (CI)**	**Fast foods Frequent**^**++ **^**use % (CI)**
Provinces	Western Cape (n = 442)	17.5 13.4 - 21.5	8.4 5.0 - 11.8	38.1 32.6 - 43.6	2.9 1.2 - 4.7
	Eastern Cape (n = 448)	22.9 18.4 - 27.3	4.9 2.5 - 7.3	19.1 15.8 - 22.4	4.7 2.1 - 7.3
	Northern Cape(n = 228)	13.2 8.2 - 18.1	1.8 0.2 - 3.3	28.9 21.4 - 36.5	2.6 0.2 - 5.1
	Free State (n = 240)	22.8 15.5 - 30.1	12.0 6.0 - 18.0	30.7 24.1 - 37.3	7.1 2.9 - 11.2
	Kwa-Zulu Natal (n = 630)	24.1 19.9 - 28.4	7.8 5.4 - 10.1	34.9 31.3 - 38.5	6.2 3.8 - 8.6
	North West (n = 136)	29.4 20.0 - 38.8	19.9 10.8 - 28.9	28.7 22.5 - 34.9	1.5 0.0 - 3.7
	Gauteng (n = 613)	20.9 17.0 - 24.8	18.9 15.3 - 22.6	38.5 34.4 - 42.6	14.7 11.2 - 18.2
	Mpumalanga (n = 246)	28.0 20.3 - 32.6	10.6 6.9 - 14.3	32.5 25.5 - 39.5	5.3 2.3 - 8.3
	Limpopo (n = 307)	26.5 20.9 - 32.0	20.6 14.2 - 27.0	27.5 21.4 - 33.5	7.5 3.6 - 11.4
	South Africa (n = 3290)	22.2 20.5 - 23.9	11.3 10.0 - 12.6	32.0 30.3 - 33.7	6.8 5.8 - 7.9

Gender	Male (n = 1338)	21.7 19.3 - 24.2	12.1 10.2 - 13.9	32.7 30.2 - 35.2	8.7 7.0 - 10.3
	Female (n = 1952)	22.6 20.5 - 24.6	10.9 9.3 - 12.4	31.5 29.3 - 33.8	5.5 4.3 - 6.8

LSM	LSM = low (n = 585)	24.1 20.3 - 27.9	9.6 6.9 - 12.2	6.2 3.9 - 8.4	0.5 0.0 - 1.1
	LSM = medium (n = 1316)	29.7 26.8 - 32.5	14.7 12.3 - 17.1	27.9 25.2 - 30.5	3.8 2.5 - 5.1
	LSM = high (n = 1218)	13.0 11.0 - 15.1	8.3 6.6 - 10.0	48.1 45.2 - 51.0	13.2 11.1 - 15.3

+Moderate use = 2-3 times a month or about once a week; ++Frequent use = 2 or more times a week

LSM = Living standards measurement; CI = 95% confidence interval

The Living Standards Measurement (LSM) system was used to classify people according to their living standards, using criteria such as degree of urbanization and ownership of cars and major appliances to place people in different categories [9].

In 2009, 3827 sampling units (projected sample) were included in the SASAS survey and a face-validated nutrition module on dietary diversity, street food intake, fast food intake and a non-quantified 24-hour recall were included in the general questionnaire [10,11]. Face validation involved four PhD dieticians checking the suitability and content (face value) of items included. The SASAS questionnaire was translated into 11 official languages of South Africa and back-translated to ensure retention of meaning. Trained interviewers completed the questionnaires while interviewing the randomly selected participants. Quality of data was assured by telephonic and physical back checks on 10% of questionnaire interviews. This ensured that corrections could be made while data collection was still in progress. These were undertaken to check that interviewers had visited the homes they were required to visit and secondly to check the correctness of data entered.

The 24-hour dietary recall included the interviewer documenting all foods and drinks consumed by the person during the previous 24 hours. A dietician checked and cleaned the final data. Mixed dishes were coded simply by entering every food item in the dish since quantities or proportions were not required. A Dietary Diversity Score (DDS) was calculated by counting each of nine food groups as they were found in the 24-hour recalls of the participants. The food group categories were the same as those used in an earlier validation study on DDS in children [12]. Each specific food item was included in one of nine selected food groups. Since national dietary data is not available on adults the children's mean DDS of 4 was used as a reference point of dietary adequacy [10,12]. A score below 4 would be indicative of poor dietary diversity while a score of nine would represent a very varied diet. A varied diet represents better food security [13,14]. Each food group was only counted once when calculating DDS. The nine groups used were- 1) cereals/roots/tubers; 2) meat/poultry/fish; 3) dairy; 4) eggs; 5) vitamin A rich fruit and vegetables; 6) legumes; 7) other fruit; 8) other vegetables; 9) fats and oils. The food items consumed were classified into 9 different groups and a dietary diversity score (DDS) was calculated for each participant with a score of 9 representing the highest DDS with the person having consumed a food item from each group A score of 1 would mean the opposite that in fact a person only consumed from one food group and hence had little variety in their diet [10,12].

The realized sample comprised 3287 adults of which 76.6% were black; 10.9% white and 12% other ethnic groups (Indian and mixed ancestry). The sample was weighted to reflect the actual population distribution. When examining frequency of consumption of fast foods and street foods, a consumption of two or more times a week was chosen to represent "*high frequency" *use, about once a week/2-3 times a month "*moderate frequency" *and seldom/never *"low frequency"*. Rationale for the use of frequency of consumption was based on two national studies in South Africa using the dietary frequency intake method [15,16].

The survey received ethics approval from the HSRC Ethics Committee. Participants signed informed consent and all information was treated confidentially. For participants who were 16 and 17 years old a signed consent was obtained from a parent or guardian whilst those 18 years and older signed their own consent.

Data was captured in Exel spread sheets and imported into the SAS software program. Data analyses included calculating frequency distributions of street food and fast foods (with 95% confidence intervals) by province, gender, ethnic group, LSM, and money spent. Average amount of money spent on street food and fast food was stratified into four categories based on local knowledge of cost of food items sold at the time of the study. Fast food and street food consumption were categorised into frequent and infrequent use and mean DDS was calculated for each category to test for adequacy of diet. Multiple logistic regression analyses of factors predicting the use of street food and fast foods was done while controlling for gender and race as confounders.

## Results

Table 1 show that frequent street food consumption was highest in Limpopo Province (20.6%), North West (19.9%), Gauteng (18.9%) and lowest in Northern Cape (1.8%). Frequent fast food consumption was highest in Gauteng (14.7%), Limpopo (7.5%), Free State (7.1%) and lowest in North West (1.5%). Adding consumption of both moderate and frequent street food shows that North West Province was the highest consumer (49.3%) while for fast food it was Gauteng (53.2%). While males and females has a similar moderate intake of street foods and fast foods, a higher percentage males consumed street food (12.1%) and fast food frequently (8.7%), compared with females (10.9 and 5.5%, respectively).

Those in the medium LSM (socio-economic) category were the most frequent buyers of street foods (14.7%) while those in the high LSM category (13.2%) were the most frequent buyers of fast foods (Table 1).

**Figure 1:**
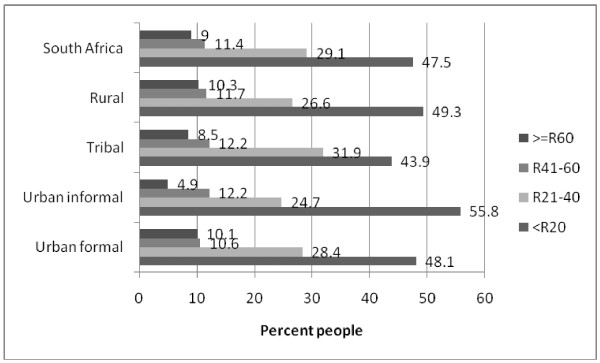
**Money spent on street food per week in different geographic areas**.

Street food were hardly used by 90.4% of whites and 84.8% of Indians in contrast to 54.7% of blacks (Table 2). 5.9% Blacks used street food every day/nearly every day compared with 3.4% of those of mixed ancestry, 2.1% of Indians and 1.7% of whites. Although the amounts are fairly small, blacks spent the most on street foods with 27.4% spending R1.0-20 ($2.90) per week and 18.8% spending R21-40 ($5.71) per week. Nearly 5% of blacks spent more than R60 ($8.57) per week (Table 2). The majority of participants spent less than R20 ($2.90) on street food per week with the most sales in this price range found in urban informal areas (55.8%) and the least in tribal areas (43.9%)(Figure 1).

**Table 2:** Percentage street food consumption by different ethnic groups in South Africa

	Street food	Black n = 1936 % (CI)	Mixed ancestry n = 604 % (CI)	Indian n = 388 % (CI)	White n = 353 % (CI)
Frequency Of use	Never/Hardly ever	54.7 52.0 - 57.4	77.6 73.5 - 81.7	84.8 80.5 - 89.0	90.4 87.0 - 93.7
	2-3 times per month	21.4 19.4 - 23.4	8.8 6.4 - 11.2	8.3 5.0 - 11.5	5.1 3.0 - 7.2
	Once a week	8.9 7.5 - 10.2	4.5 2.9 - 6.1	2.8 1.1 - 4.6	1.4 0.2 - 2.7
	2-3 times per week	9.1 7.8 - 10.4	5.8 3.7 - 8.0	2.1 0.5 - 3.6	1.4 0.2 - 2.6
	Nearly every day	3.9 3.0 - 5.0	1.7 0.7 - 2.6	1.3 0.2 - 2.4	0.6 0.0 - 1.3
	Every day	2.0 1.3 - 2.8	1.7 0.5 - 2.8	0.8 0.0 - 1.6	1.1 0.0 - 2.5

Money spent on street food per week	R0	41.3 38.6 - 43.9	60.9 56.0 - 65.9	75.8 70.7 - 81.0	82.4 78.0 - 86.8
	R1 - R20	27.4 25.2 - 29.6	18.5 15.2 - 21.9	11.1 7.5 - 14.6	8.8 5.7 - 11.9
	R21 - R40	18.8 17.0 - 20.6	13.4 10.6 - 16.2	7.5 4.8 - 10.1	5.1 2.7 - 7.5
	R41 - R60	6.3 5.0 - 7.5	3.1 1.7 - 4.6	3.1 1.3 - 4.9	2.5 0.7 - 4.4
	> R60	4.6 3.5 - 5.8	2.6 1.3 - 4.0	2.3 0.9 - 3.7	1.1 0.0 - 2.2
	Don't know	1.6 0.9 - 2.3	1.3 0.3 - 2.3	0.3 0.0 - 0.8	-

Type of Street food	Fruit	35.8 33.3 - 38.2	20.0 16.3 - 23.8	13.9 9.8 - 18.0	9.6 6.4 - 12.9
	Soft drinks	16.4 14.6 - 18.3	13.4 10.3 - 16.5	8.7 5.7 - 11.8	4.8 2.2 - 7.4
	Cooked food	9.7 8.3 - 11.2	4.8 3.0 - 6.6	3.1 1.2 - 5.0	1.1 0.0 - 2.2
	Sweets & biscuits	11.8 10.2 - 13.4	5.8 3.7 - 7.9	2.6 1.1 - 4.0	1.7 0.4 - 3.0
	Savoury snacks	14.5 12.7 - 16.2	11.6 9.0 - 14.2	7.2 4.7 - 9.7	2.3 0.8 - 3.8
	Other	13.1 11.4 - 14.7	9.6 6.6 - 12.6	4.4 2.3 - 6.4	5.1 2.4 - 7.8

CI = 95% confidence interval

Overall, fruit was the most commonly purchased street food by all ethnic groups over the previous week although this practice was highest in black participants (35.8%)(Table 2). Purchases of soft drinks ranged from 4.8% in whites to 16.4% in blacks and savoury snacks from 2.3% to 14.5% in whites and blacks, respectively.

In contrast to street foods, 67.6% blacks and 60.5% mixed ancestry participants hardly ever purchased fast foods, while this decreased to less than 45% in Indians and whites (Table 3). The majority of participants in all ethnic groups consumed fast foods 2-3 times a month. The largest percentage black (23.4%) and mixed ancestry participants (25.7%) spent between R26-75 ($10.71) per month while the highest percentage Indians (23.1%) and whites (21.2%) spent between R101-200 ($28.57) per month. 14.4% Whites and 15.2% Indians spent more than R200 ($28.57) per month on fast foods.

**Table 3:** Percentage fast food consumption by different ethnic groups in South Africa

	Fast food	Black n = 1938 % (CI)	Mixed ancestry n = 601 % (CI)	Indian n = 389 % (CI)	White n = 353 % (CI)
Frequency Of use	Never/Hardly ever	67.6 65.2 - 70.2	60.5 56.2 - 64.8	43.4 38.4 - 48.5	44.8 38.7 - 50.8
	2-3 times per month	21.4 19.4 - 23.5	26.8 23.0 - 30.5	30.1 25.7 = 34.4	28.3 23.6 - 33.1
	Once a week	5.5 4.5 - 6.6	7.4 5.3 - 9.4	13.9 10.6 - 17.1	15.6 11.9 - 19.3
	2-3 times per week	4.3 3.2 - 5.4	3.8 2.3 - 5.4	9.3 6.2 - 12.3	8.2 5.3 - 11.2
	Nearly every day	0.8 0.4 - 1.2	0.8 0.1 - 1.6	1.8 0.4 - 3.2	2.8 0.9 - 4.8
	Every day	0.2 0.0 - 0.4	0.7 0.0 - 1.3	1.5 0.4 - 2.7	0.3 0.0 - 0.8

Money spent on fast food per month	R0	50.9 48.2 - 53.5	34.8 29.8 - 39.7	19.0 14.9 - 23.1	25.2 19.2 - 31.2
	R1 - R25	5.9 4.8 - 7.0	8.3 5.9 - 10.7	3.6 1.6 - 5.6	4.8 2.7 - 7.0
	R26 - R75	23.4 21.2 - 25.5	25.7 21.4 - 29.9	22.4 17.1 - 27.6	17.8 13.2 - 22.5
	R76 - R100	7.5 6.3 - 8.6	13.2 10.3 - 16.2	13.6 10.4 - 16.9	15.9 11.6 - 20.1
	R101-R200	7.3 6.0 - 8.5	11.1 8.1 - 14.1	23.1 18.6 - 27.7	21.2 16.9 - 25.6
	> R200	4.3 3.3 - 5.4	5.8 3.8 - 7.8	15.2 10.5 - 19.9	14.4 10.6 - 18.3
	Dont know	0.8 0.3 - 1.2	1.2 0.2 - 2.1	3.1 1.2 - 4.9	0.6 0.0 - 1.3

CI = 95% confidence interval

Table 4 evaluates the effect of different factors on frequent street food and frequent fast food use. The odds of purchasing fast food frequently was highest when people were employed (Odds Ratio = 1.53; CI 1.01-2,31); lived in a formal house (OR = 2.75; CI = 1.12-6.74) with water (OR = 2.96; CI 2.16-4.06) and electricity (OR = 2.07; CI 1.13-3.80) in the home. Possession of home assets such as fridge, freezer and microwave all also had significant OR values. Having a fridge with freezer, microwave oven, electric stove, TV, motor vehicle and DVD player increased odds up to 4 times for frequent fast food purchasing. Odds ratios for street foods were similar to those for fast food purchasing albeit lower. Table 5 illustrates that frequent fast food consumption resulted in a significantly higher DDS than those who seldom purchased fast food (4.69 vs. 3.73; p < 0.0001). On the other hand frequent street food purchasing resulted in a significantly lower DDS (3.81 vs 4.13; p < 0.0001) than those who purchased it seldom.

**Table 4:** Logistic regression of factors which impact on frequent (2 or more times a week) purchasing of street food and fast foods adjusted for race and gender

	Frequently buy street food	Frequently buy fast food
	Odds Ratio	Odds Ratio
**Employment status**		
Employed full time	NS	1.53* [1.01 - 2.31]
Employed part time	NS	
Casual work	NS	0.29* [0.13 - 0.63]
Unemployed	NS	NS
Disabled	NS	0.33* [0.15 - 0.76]
**Income**		
Receive salary/wages	NS	NS
Remittances	NS	0.58* [0.35 - 0.97]
Pensions and/or grants	0.58* [0.37 - 0.88]	0.35* [0.24 - 0.50]
**Where usually buys**		
Local spaza	NS	0.40* [0.22 - 0.73]
Big supermarket close by	NS	NS
Big supermarket far away	NS	NS
**Housing**		
Formal: house/apartment	NS	2.75* [1.12 - 6.74]
Traditional dwelling	NS	NS
Townhouse	NS	3.39* [1.32 - 8.70]
Informal dwelling	NS	NS
**Water**		
Water in home	1.65* [1.11 - 2.44]	2.96* [2.16 - 4.06]
Water in yard	NS	NS
Free communal tap	NS	0.56* [0.37 - 0.84]
Free from neighbour	NS	0.15* [0.04 - 0.64]
Communal borehole	0.54	0.36* [0.14 - 0.97]
River, stream or spring	0.32* [0.12 - 0.85]	0.08* [0.02 - 0.35]
**Toilet**		
All types	NS	NS
None	NS	NS
**Electricity**		
In-house meter	NS	2.07*[1.13 - 3.80]
No access	NS	0.22*[0.11 - 0.44]
**Other assets**		
Fridge with freezer	1.42* [1.15 - 1.75]	4.62* [3.70 - 5.75]
Deep freezer	1.34* [1.07 - 1.67]	2.34* [1.98 - 2.77]
Microwave	1.42* [1.17 - 1.73]	4.76* [3.98 - 5.69]
VCR	NS	1.84* [1.53 - 2.20]
Electric stove	1.28* [1.03 - 1.60]	4.31* [3.43 - 5.42]
TV	1.55* [1.23 - 1.96]	4.17* [3.28 - 5.29]
Telephone at home	NS	2.05* [1.71 - 2.45]
Kitchen sink	NS	3.76* [3.13 - 4.51]
Home security	NS	3.39* [2.74 - 4.17]
Motor vehicle	NS	4.45* [3.72 - 5.32]
Mobile phone	1.54* [1.19 - 1.98]	3.60* [2.81 - 4.60]
Music centre	NS	2.36* [2.02 - 2.75]
DVD player	1.67* [1.37 - 2.03]	4.32* [3.62 - 5.17]

**Table 5:** Comparison of dietary diversity score with frequent, moderate and infrequent consumption of street food and fast food

	Mean DDS	95% CI	Kruskal-Wallis p-value
**Street foods**			

Never or Hardly ever (n = 2171)	4.13[A]	4.07 - 4.20	p < 0.0001**
Moderate+ (n = 730)	3.77[B]	3.66 - 3.89	
Frequent++ (n = 373)	3.81[B]	3.66 - 3.96	

**Fast foods**			

Never or Hardly ever (n = 1999)	3.73[C]	3.66 - 3.80	< 0.0001**
Moderate+ (n = 1052)	4.41[B]	4.32 - 4.50	
Frequent++ (n = 224)	4.69[A]	4.51 - 4.87	
All (n = 3287)	4.02	3.96 - 4.07	

+Moderate use = 2-3 times a month or about once a week; ++Frequent use = 2 or more times a week

**p < 0.0001 = significant difference in DDS between frequent and seldom consumers

[A], [B], [C] Different symbols indicate significant differences amongst groups, Bonferroni multiple comparison test, significant for p < 0.05.

## Discussion

The results of this survey showed that street food and fast food consumption are commonly consumed in all provinces of South Africa and consequently contribute to dietary intake, particularly those who consume it frequently. A recent pilot survey [17] in deep rural villages in Mpumalanga showed similar trends, with the sale of street foods being a very common occurrence even in these very rural and remote areas.

The increasing use of fast foods, and more recently street foods, pose many questions for public health advocates. Should nutritionists condemn the use of street foods in a country which is undergoing a rapid transition from consumption of traditional foods to more commercially available, ready-to-eat foods? How do these foods "fit" into the food-based dietary guidelines [18] which health professionals in South Africa promote? Apart from fruit, which was found to be a popular street food choice, the remaining items would not be nutritionally recommended by nutritionists and would not feature on the list of food-based dietary guidelines. However, the reality lies in the fact that street foods are generally very affordable [2,17] and have a high energy density. As such they are very appealing to poor people who need to reduce their hunger as cheaply as possible.

In this study, apart from the documentation of food items selected, we have little information on the most commonly consumed "cooked" fast foods and street foods in terms of their nutritional value or portion sizes. One of the limitations of the present study was that cooked foods were not individually identified and due to the nature of the study, portion sizes could not be calculated. This appears to be a large gap in the nutrition knowledge that we have on the dietary intake of adults in South Africa and motivates for the importance of a national food consumption survey in the adult population. The pilot study undertaken in Mpumalanga [17] showed that items most commonly available for sale in those areas were fried potato chips, vetkoek (fried dumplings made from wheat flour) and kotas (a quarter loaf of white bread filled with fried potato chips, and numerous processed meat or cheese). The fried chips and vetkoek yielded on average between 943 and 5552 kJ and between 11-64 g fat, being high in energy and in fat.

Another aspect of critical importance in the discussion around the relative merits of fast food consumption are the negative nutritional outcomes [19-24] associated with its use; while there are limited data in this regard in relation to the consumption of street foods. One of the negative aspects relates to the association of fast food consumption with overweight and obesity, both of which are very high in South African women (73% have a BMI > = 25)[4]. The high prevalence of fast food and street food intake, particularly intake of soft drinks is of some concern because of the recent flurry of studies documenting the positive relationship between soft drink consumption and obesity in children and adolescents [25-30]. In South Africa, as in many other African countries, there is little information regarding the extent to which use of street foods or fast foods contribute to this problem. However, based on the results from the Mpumalanga study [17] and the Soweto study [2], one can only assume that similar findings will prevail in many other areas. This is certainly an aspect that should be investigated in a future research agenda and is currently receiving attention in the development of a protocol for a national nutrition study in South Africa. Another limitation of the present study was the fact that types of fast foods consumed were not recorded.

The present study showed that socio-economic status played an extremely important role in the consumption of both fast foods and street foods, particularly in relation to fast food intake. Fast food intake was much higher in the highest LSM compared with the low LSM, while those in the middle category were the highest consumers of street foods. The importance of socio-economic status was further illustrated by the findings of the logistic regression. People who were employed, and additionally possessed major household appliances had a higher intake of fast foods; reflecting the effects of westernization of diet in Africa. This was also the case for street foods, however, to a lesser degree, most probably because street foods were less expensive and there was less variety in the items sold.

Furthermore, another aspect of street foods that requires consideration is the convenience aspect. People who live far from their place of employment, as is the case around the big cities, and have to travel long distances may be in a situation where it is difficult for them to have regular meals at home. In such cases a source of food already prepared, immediately accessible, and at low cost will meet their immediate needs. It is also necessary to consider the finding that those having a frequent intake of fast foods had a significantly higher dietary diversity than those who did not, while those who had a frequent street food intake had a significantly lower dietary diversity than those who seldom consumed street foods. This implies that there was less diversity in the diet of those purchasing street foods more frequently and hence can be considered a serious disadvantage in terms of food security [14]. Street food should therefore be seriously investigated from a policy point of view in terms of their potential impact on food security.

The last aspect of street food that has to be mentioned is one on food hygiene and safety, due to the large numbers of people who purchase such foods and the possible health risks involved. Microbial studies conducted on street foods in both Bloemfontein and Johannesburg have found that the safety of street foods was better than expected in these two urban areas studied [31-33]. Two critical points were identified as ensuring best safety of foods including cooking at temperatures over 65 degrees C and having short holding times. The practices studied in Johannesburg indicated that vendors bought food from retailers early every day; prepared the food in sufficient quantities for that day and gave away or took home the leftovers. This practice is thought to have contributed to the reasonably safe microbial content of the street foods examined but may have had ramifications for the families at home who most probably eat the left-overs. Von Holy et al. have stressed the importance of a need for running water and toilets in areas where street vendors operate [34]; however, there does not appear to be national data on the food safety aspects of such enterprises. We would like to recommend that this aspect is given serious consideration when the next national survey is planned since the extent of street food consumption in RSA certainly warrants this.

Lastly, the consumption of street food provides employment for a large sector of the population [1] and there may be ways and means by which this practice can be encouraged if vendors sell healthier food items; such as fresh fruit, dry fruit, nuts, and vegetables such as roasted maize cobs. Since the sale of street food is likely to increase, consideration should be given to ways and means that the practice can be done in a healthy and safe manner. This is particularly relevant in schools in poorer areas where at times numerous vendors sell their products to children at break periods [2]. The bulk of these items are energy-dense and high in fat, already laying a foundation for the type of diet which one wishes to avoid in countries which are undergoing the nutrition transition.

In conclusion, a large percentage of the population purchases fast foods and street foods. This is of some concern when one notes the high prevalence of soft drinks, high fat and energy-dense foods consumed, particularly in terms of pre-disposition to obesity, and related chronic diseases. These findings need to be taken into consideration when evaluating and planning dietary patterns and nutritional adequacy of population diets and health promotion intervention programs.

## Competing interests

The authors declare that they have no competing interests.

## Authors' contributions

NPS and DL: conceptualized paper; wrote and reviewed the manuscript. JHN: data analysis and interpretation. All authors read and approved the final manuscript.
